# Relationship Analysis of Central Venous-to-arterial Carbon Dioxide Difference and Cardiac Index for Septic Shock

**DOI:** 10.1038/s41598-019-45252-6

**Published:** 2019-06-19

**Authors:** Zhiqiang Guo, Ming Yin, Jichang Kong, Bin Wang, Kunpeng Dai, Tian Zuo, Guangyan Yu, Yong Bao

**Affiliations:** 1 Department of Critical Care Medicine of Beidaihe Hospital of Qinhuangdao, Qinhuangdao, China; 2 Department of Interventional Endoscopy of General Hospital of North China Petroleum Administration, Renqiu, China; 3 Deparment of Critical Care Medicine of General Hospital of North China Petroleum Administration, Renqiu, China

**Keywords:** Health care, Disease-free survival

## Abstract

To analyze the relationship of the central venous-to-arterial carbon dioxide difference (*p*(cv-a)CO_2_) and cardiac index (CI) in patients with septic shock, an observational study was conducted in intensive care unit (ICU). 66 consecutive patients with septic shock and central venous oxygen saturation (ScvO_2_) ≥ 70% were included after early fluid resuscitation. Measurements were taken at a 6 h interval (T0, T6, T12, T18, T24) during first 24 h after their admission into ICU, including heart rate (HR), mean arterial pressure (MAP), central venous pressure (CVP), *p*(cv-a)CO_2_, cardiac index(CI, L/(min•m^2^)) and ScvO_2_. Patients were divided into low *p*(cv-a)CO_2_ group (n = 35) and high *p*(cv-a)CO_2_ group (n = 31) according to a threshold of 6 mmHg for *p*(cv-a)CO_2_ at T0. As a result, at T0, T6, T12, T18 and T24, there were respectively significant differences between low and high *p*(cv-a)CO_2_ groups for CI (4.1 ± 1.4 vs 2.4 ± 0.6, 4.4 ± 0.9 vs 2.8 ± 0.7, 4.1 ± 1.3 vs 2.9 ± 0.6, 4.0 ± 1.3 vs 2.7 ± 0.8, 4.2 ± 1.4 vs 2.9 ± 0.8, *p* < 0.001 at each time point), 28-day mortality rate was 38.7%(12/31) for high *p*(cv-a)CO_2_ group and 22.8% (8/35) for low *p*(cv-a)CO_2_ group (*p* > 0.05), there were significant differences for *p*(cv-a)CO_2_ (*p* < 0.05) between low and high *p*(cv-a)CO_2_ groups, no differences for HR, MAP, CVP, ScvO_2_ (*p* > 0.05). CI was inversely correlated with *p*(cv-a)CO_2_ value (*r* = −0.804, *p* < 0.001), but not for ScvO_2_(*r* = 0.08, *p* > 0.05). Receiver operating characteristic curve analysis confirmed the correlation of *p*(cv-a)CO_2_ with CI (AUC: 0.782;*p* < 0.001; 95% confidence interval: 0.710–0.853). The cut-off value for the best predictive value of CI ≥ 2.2 L/(min·m^2^) was *p*(cv-a)CO_2_ of 5.55 mmHg or lower with a sensitivity of 85.7% and specificity of 66.8%. Hence CI measured with USCOM is inversely correlated with *p*(cv-a)CO_2_ values in guiding the resuscitation of patients with septic shock.

## Introduction

In 2016, sepsis and septic shock was re-documented as fatal organ dysfunction caused by infection-induced host response disorders^1^. Infectious shock is a subtype of sepsis; its circulation abnormalities significantly increase the mortality rate. Low blood volume, antihypertensive drugs needed to maintain MAP ≥ 65 mmHg and serum lactic acid >2 mmol/L can confirm septic shock. The definition was updated to facilitate rapid identification and timely treatment. Despite the continuous progress of awareness and intervention, the mortality rate of septic shock is approaching 40% or more^2^. Infectious shock exists in the presence of imbalance of oxygen supply and demand as well as tissue hypoxia, early improvement of tissue hypo-perfusion is key to the treatment, a specific cluster treatment program was recommended in the guidelines of sepsis rescue action^3^. Central venous oxygen saturation as one of the key goals of early goal management is widely used to guide resuscitation therapy for better tissue perfusion. In fact, normal ScvO_2_ does not exclude tissue hypo-perfusion^4,5^. Carbon dioxide partial pressure difference is proportional to carbon dioxide production, and is inversely proportional to cardiac output, its normal value is 2~5 mmHg, CO of more than 6 mmHg means that peripheral blood flow is not sufficient to clear out carbon dioxide^6^. Numerous studies suggest that partial pressure difference of carbon dioxide may be an indicator to improve tissue perfusion for guiding early resuscitation of sepsis^7–9^. The use of continuous ultrasonic cardiac output monitor (USCOM), which is noninvasive and widely used in the severely sick patients, is convenient, fast and reproducible to monitor hemodynamics and to guarantee the accuracy of cardiac output and cardiac index^10^. This study was conducted to investigate the relationship between (*p*(cv-a)CO_2_) and USCOM-measuring CI in patients with septic shock.

## Patients and Methods

Jan 2016 Through Dec 2017, 66 patients with septic shock were admitted into the severe medical department of Beidaihe Hospital and North China Petroleum Administration General Hospital (ICU), those patients who undertook early fluid resuscitation in 6 hours and got the value of ScvO_2_ ≥ 70% were included in this retrospective study following the criteria: (1) met the diagnostic criteria of septic shock issued by international sepsis and septic shock treatment guidelines for septic shock in 2011^3^; (2) early fluid resuscitation was conducted following the cluster treatment program of the sepsis action guideline. According to the guideline, the early goal-oriented treatment was applied in our unit. (3) ScvO_2_ ≥ 70% was achieved 6 hours after resuscitation; (4) ≥18 years old. Patients with incomplete data were excluded. Baseline venous and arterial blood gas analysis was measured every 6 hours in 24 hours. USCOM was used to measure CI, its probe was placed on the sternum or the supra-clavicular fossa to obtain the strongest signal, and measurement was taken for 3 consecutive times, each deviation did not exceed 10% to take the average of CI. This study was approved by Ethics Committees of Baidaihe Hospital and North China Petroleum Hospital. Informed consents were obtained from the patients’ family. All procedures followed were in accordance with the ethical standards of responsible committee on human experimentation (institutional and national) and with the Helsinki Declaration of 1964 and later versions.

The patients’ age, sex, diagnosis, APACHE IIscore were obtained. Early fluid resuscitation was started, time points of 0, 6, 12, 18, 24 hours after resuscitation were T0, T6, T12, T18, T24. Data like heart rate (HR), MAP, CVP, *p*(cv-a)CO_2_, CI and ScvO_2_ was collected at each time point. *p*(cv-a)CO_2_ was the difference between *p*cvCO_2_ and *p*aCO_2_, the normal value was less than 6 mmHg. The cut-off value of *p*(cv-a)CO_2_ at T0 was 6 mmHg, the group with <6 mmHg or with ≥6 mmHg were low *p*(cv-a)CO_2_ group (T0) or high *p*(cv-a)CO_2_ group. Based on the hemodynamic data, the relationship between CI and *p* (cv-a)CO_2_ was analyzed.

### Statistical

All numeration variables were stated as median, and continuous variables were presented as means ± standard deviation and tested by normal distribution and analyzed by One-Way ANOVA; sampling rates were analyzed with x^2^ test, the correlation between CI and *p*(cv-a)CO_2_ and ScvO_2_ was analyzed by the Spearman test, the diagnostic characteristic curve (ROC curve) was used to evaluate the diagnostic value of the parameters, the value of *p* < 0.05 was set as significant difference. All data was analyzed by statistical software SPSS22.0 (IBM Corp, Armonk, NY, USA).

## Results

**Figure 1:**
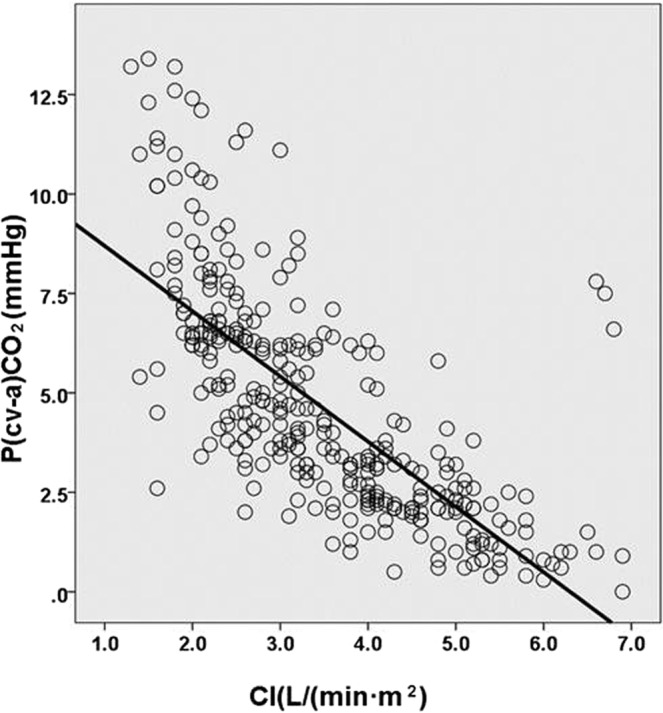
Correlation of CI and *p*(cv-a)CO_2_, R^2^ = 0.536, *p* < 0.001.

**Figure 2:**
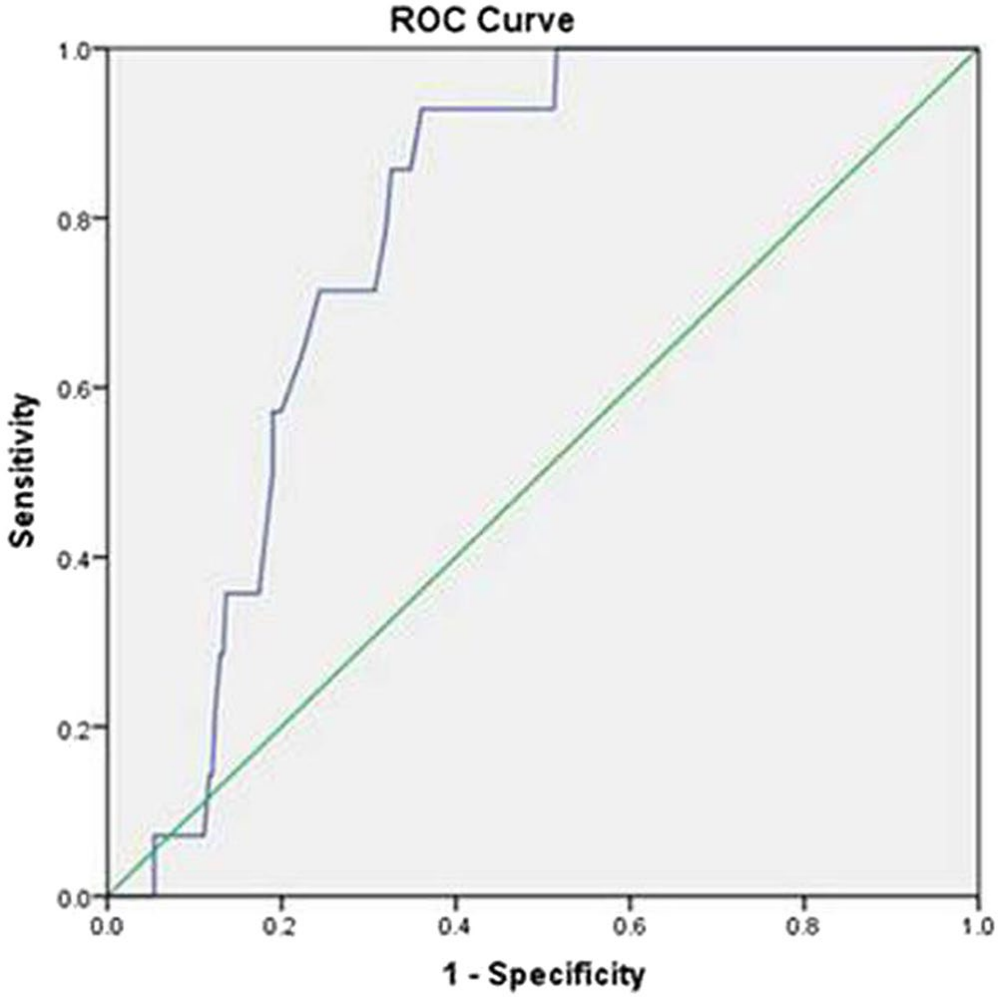
ROC curve for *p*(cv-a)CO_2_ to predict CI with >2.2 L/(min·m^2^), Area under ROC curve was 0.782, the sensitivity was 0.857 and the specificity was 0.668.

For the low *p*(cv-a)CO_2_ group, 20 male and 15 female patients aged 64.9 ± 12.5 years, the number of patients with ScvO_2_ ≥ 70% at T0 was 24 (68.6%). For the high *p*(cv-a)CO_2_ group, 18 male and 13 female patients aged 65.5 ± 10.4 years, the number of patients with ScvO_2_ ≥ 70% at T0 was 16(51.6%). At T6, the number of patients with *p*(cv-a)CO_2_ ≥ 6 mmHg in the low *p* (cv-a)CO_2_ group was 3(8.6%), while this number in the high *p*(cv-a)CO_2_ group was 17(54.8%), the total number of patients with *p*(cv-a)CO_2_ ≥ 6 mmHg was 20, account for 30.3% of all patients. There were no significant differences in age, sex and APACHEIIscore between two groups (*p* > 0.05) (Table 1), and no difference for patients with ScvO_2_ ≥ 70% (*p* > 0.05), no difference for infection sites including lung, peritoneal cavity, urinary system and other sites. For HR, MAP, CVP, there were no significant differences between two groups (*p* > 0.05), 28-day mortality rate was different, which was 38.7% (12/31)for high *p*(cv-a)CO_2_ group and 22.8% (8/35) for low *p*(cv-a)CO_2_ group (*p* > 0.05). There were significant differences for CI at respective time point between two groups (*p* < 0.001) (Table 2). The value of *p*(cv-a)CO_2_ was evaluated by ROC curve to predict CI (Fig. 1). Area under ROC curve was 0.782, the sensitivity was 0.857 and the specificity was 0.668 when *p*(cv-a)CO_2_ was ≤5.55 mmHg to follow CI ≥ 2.2 L/(min•m^2^) (Fig. 2).

**Table 1:** Characteristics of patients at T0.

	Low *p*(cv-a)CO_2_ (n = 35)	High *p*(cv-a)CO_2_ (n = 31)	*p*
Age (years)	64.9 ± 12.5	65.5 ± 10.4	0.827
Gender(male/female)	20/15	18/13	0.941
BMI(kg/m^2^)	21.4 ± 6.1	23.4 ± 7.1	0.079
ScvO_2_ ≥ 70%(%)	24 (68.6)	16 (51.6)	0.209
APACHE II	21.7 ± 4.3	21.8 ± 3.2	0.901
**Diagnosis n (%)**			
Pneumonia	23(66)	21(68)	0.862
Abdominal infection	6(17)	5(16)	0.912
Urinary tract infection	2(6)	2(6)	0.900
Others	4(11)	3(10)	0.818
HR^*^(beats/min)	107	113	0.761
MAP(mmHg)	56 ± 8	54 ± 8	0.221
CVP(mmHg)	5 ± 2	4 ± 3	0.075
CI(L/min·m^2^)	4.1 ± 1.4	2.4 ± 0.6	<0.001
*p*(cv-a)CO_2_/mmHg	3.3 ± 1.3	7.9 ± 1.9	<0.001
ScvO_2_(%)	71 ± 6	69 ± 5	0.096
28-day mortality rate	22.8% (8/35)	38.7% (12/31)	>0.05

BMI: body mass index, APACHE: acute physiology and chronic health evaluation, HR: heart rate, MAP: mean arterial pressure, CVP: central venous pressure, CI: cardiac index, *p*(cv-a)CO_2:_ central venous-arterial carbon dioxide difference, ScvO_2:_ central venous oxygen saturation. HR* presented as median.

**Table 2:** Hemodynamic parameters at each time point.

		Low	*p* (cv-a)	CO_2_			High	*p* (cv-a)	CO_2_	
	T0	T6	T12	T18	T24	T0	T6	T12	T18	T24
HR	122	108	100	98	110	122	114	110	110	106
p*	0.761	0.111	0.107	0.205	0.060					
MAP(mmHg)	56 ± 8	73 ± 10	78 ± 12	81 ± 15	84 ± 17	54 ± 8	69 ± 13	73 ± 10	76 ± 11	77 ± 12
p*	0.221	0.155	0.129	0.123	0.062					
CVP (mmHg)	5 ± 2	10 ± 3	10 ± 3	10 ± 3	10 ± 3	4 ± 3	9 ± 3	9 ± 3	9 ± 3	10 ± 3
p*	0.075	0.197	0.172	0.517	0.677					
CI (L/min.m^2^))	4.1 ± 1.4	4.4 ± 0.9	4.1 ± 1.3	4.0 ± 1.3	4.2 ± 1.4	2.4 ± 0.6	2.8 ± 0.7	2.9 ± 0.6	2.7 ± 0.8	2.9 ± 0.8
p*	<0.001	<0.001	<0.001	<0.001	<0.001					
ScvO_2 (%)_	71 ± 6	75 ± 3	74 ± 6	73 ± 6	75 ± 4	69 ± 5	74 ± 4	73 ± 5	75 ± 5	75 ± 5
p*	0.096	0.298	0.321	0.183	0.684					

p*: parameters compared respectively at each time point.

## Discussion

*p*(cv-a)CO_2_ is well-accepted to study carbon dioxide partial pressure difference^8^, the combination of *p*(cv-a)CO_2_ and ScvO_2_ can guide therapeutic shock resuscitation to avert fluid overload due to pseudo-normalization of ScvO_2_. The clinical significance of *p*(cv-a)CO_2_ is as follows: (1) The initial increase of *p*(cv-a)CO_2_ is more than 6 mmHg, which indicates that the blood flow may be insufficient, even if the microcirculation parameters (including ScvO_2_) are normal. CO should be increased to improve tissue perfusion, especially in the presence of hypoxia (elevated lactate). (2) normal *p*(cv-a)CO_2_ range (<6 mmHg) is indicative that blood flow from the peripheral cycle clears out carbon dioxide, improving heart output is not the preferred treatment, even if tissue hypoxia exists^9^. In our study, under the condition of ScvO_2_ compliance after resuscitation, most patients achieved *p*(cv-a)CO_2_ ≥ 6 mmHg at T0, T6. Although after the early fluid resuscitation treatment, there are still some patients with insufficient venous blood flow to clear out CO_2_ in the peripheral tissue. In the early resuscitation phase, those patients may need more active and longer duration of resuscitation therapy. Leisman *et al*. reported that fluid resuscitation was started in these patients in 30 min, the mortality rate was only 13.3%, and that initiation of fluid resuscitation was delayed 30 min, mortality increased over the prolonged fluid resuscitation^10–13^. Numerous studies have shown that *p*(cv-a)CO_2_ can be one of the goals of resuscitation treatment. There were no significant differences in hemodynamic parameters respective of CI and *p*(cv-a)CO_2_ between two groups in our study, in the process of resuscitation, cardiac output is clinically lower than normal, ScvO_2_ cannot be low. When shock occurs, mitochondria cannot use oxygen for energy metabolism, it is cytopathic hypoxia, although this stage ScvO_2_ can be increased, or even abnormally increased, unrelated to more oxygen transport, it is still an important manifestation of tissue hypoxia that predicts a poor prognosis^4^. CI is conveniently measured with USCOM, which is a new non-invasive hemodynamic monitoring method that is continuous Doppler technology to obtain hemodynamic parameters. Compared with floating catheters and pulse-indicated continuous cardiac output with invasive operations and catheter-related complications^14^, USCOM is increasingly applied in the clinical practice due to its rapidity and reproducibility to monitor hemodynamics and to guarantee the accuracy of CO and CI. *p*(cv-a)CO_2_ is inversely proportional to CO, and *p*(cv-a)CO_2_ is elevated in case of tissue CO_2_ retention when cardiac function is not good enough to provide adequate venous blood flow. Our report demonstrated that CI in some patients was still low after successful resuscitation. Rivers *et al*. reported that the outcomes for an early goal-oriented treatment for sepsis and septic shock, mortality rate was decreased by 15% of patients^15–17^. Ho *et al*. reported that the application of key point control strategies improved the compliance of clinicians with guidelines for cluster therapy for septic shock, the ICU stay time was reduced from 9.8 days to 7.2 days, in-hospital mortality rate went down from 40.0% to 23.1%^12^, our study substantiated that *p*(cv-a)CO_2_ was negatively correlated with CI, not correlated with ScvO_2_. Valle’e *et al*. reported that 6 mmHg as the threshold was predictive of insufficient early resuscitation for patients to reach ScvO_2_ ≥ 70%^18–20^. Mallat *et al*. also reported that CI and *p*(cv-a)CO_2_ were negatively correlated^13^, our result resembled these documents though our limitation in this report was mainly due to small sample size. Low CI means that venous blood flow is not sufficient to wash out CO_2_ in the peripheral tissue^14^, Therefore, simultaneous monitoring of CI and *p*(cv-a)CO_2_ is beneficial to determine cardiac function status and whether adequate venous blood flow is provided to meet the need of CO_2_ clearance from peripheral tissues.

## Conclusion

In sepsis, CI and *p*(cv-a)CO_2_ are negatively correlated, the latter is useful parameter to evaluate cardiac output in the course of shock resuscitation.
